# A Multi-Study Exploration of Factors That Optimize Hardiness in Sport Coaches and the Role of Reflective Practice in Facilitating Hardy Attitudes

**DOI:** 10.3389/fpsyg.2020.01823

**Published:** 2020-08-13

**Authors:** Brendan Cropley, Lee Baldock, Sheldon Hanton, Daniel F. Gucciardi, Alan McKay, Rich Neil, Tom Williams

**Affiliations:** ^1^School of Health, Sport and Professional Practice, University of South Wales, Pontypridd, United Kingdom; ^2^School of Sport and Health Sciences, Cardiff Metropolitan University, Cardiff, United Kingdom; ^3^School of Physiotherapy and Exercise Science, Curtin University, Perth, WA, Australia

**Keywords:** sport coaching, hardiness, experiential learning, reflective practice, mixed-methods, latent profile analysis

## Abstract

Hardiness has been identified as a key personal characteristic that may moderate the ill-effects of stress on health and performance. However, little is known about how hardiness might be developed, particularly in sport coaches. To systematically address this gap, we present two linked studies. First, interviews were conducted with pre-determined high-hardy, elite coaches (*n* = 13) to explore how they had developed their hardy dispositions through the associated attitudinal sub-components of control, commitment, and challenge. Utilizing thematic analysis, we identified that hardiness was developed through experiential learning, external support, and the use of specific coping mechanisms. Key to all of these themes was the concept of reflective practice, which was thought to facilitate more meaningful learning from the participants’ experiences and, subsequently, enhance the self-awareness and insight required to augment hardiness and its sub-components. To investigate further the potential relationship between coaches’ reflective practices and their level of hardiness, we conducted a follow-up study. Specifically, a sample of 402 sports coaches completed the Dispositional Resilience Scale-15, the Self-Reflection and Insight Scale, and the Questionnaire for Reflective Thinking. Using latent profile analysis (LPA), we clustered participants into groups based on their reflective profiles (e.g., type of engagement, level of reflective thinking). We then examined differences in hardiness between the five latent sub-groups using multinomial regression. Findings revealed that the sub-group of *highly engaged, intentionally critical reflective thinkers* reported significantly higher levels of all three hardiness sub-components than all other sub-groups; these effect sizes were typically moderate-to-large in magnitude (standardized mean differences = −1.50 to −0.10). Conversely, the profile of *highly disengaged, non-reflective, habitual actors* reported the lowest level of all three dimensions. Collectively, our findings offer novel insights into the potential factors that may influence a coaches’ level of hardiness. We provide particular support for the importance of reflective practice as a meta-cognitive strategy that helps coaches to develop hardy dispositions through augmenting its attitudinal sub-components. Consequently, our research makes a significant contribution by providing a comprehensive insight into how we might better train and support coaches to demonstrate the adaptive qualities required to thrive in demanding situations.

## Introduction

Sports coaching has become widely recognized as an inherently stressful profession in which individuals encounter a range of organizational, competitive, and personal stressors due to the complex multiplicity of their role (Thelwell et al., 2008; Potts et al., 2018; Cropley et al., 2020b). Couple this with (amongst many other factors) increasing internal and external pressures to perform (McNeill et al., 2018), long working hours (Knight et al., 2013), the volatile nature of the sport environment (Hill and Sotiriadou, 2016), increased scrutiny (Fletcher and Scott, 2010), and high levels of job insecurity (Carson et al., 2019), it is unsurprising that coaches may experience reduced performance effectiveness (cf. Thelwell et al., 2017). Further, a growing body of research has reported that the nature of sport coaching has the potential to decrease professional functioning and detrimentally impact on physical health and mental well-being outcomes, including emotional exhaustion (e.g., Olusoga et al., 2019), depression (e.g., Didymus, 2017), and burnout (e.g., Bentzen et al., 2017). However, while some coaches experience these negative outcomes, others show more adaptive response patterns, and even thrive (e.g., experience of high levels of well-being and performance; cf. Brown et al., 2017) in the high-pressure coaching environment (McNeill et al., 2018). What distinguishes between those coaches who suffer when experiencing stress and related mental well-being outcomes, compared to others that show remarkable resilience, often remaining physically and mentally healthy despite high levels of demand, is of particular interest to researchers, sports psychologists, and coach educators alike (Cropley et al., 2020b).

To date, our understanding of coach stress and associated outcomes has predominantly been developed through the use of Lazarus and Folkman’s (1984) transactional theory of stress and Lazarus’s (1999) cognitive-motivational-relational theory (for a review see Norris et al., 2017). These perspectives posit that stress is a dynamic and ongoing transaction between environmental demands (i.e., stressors), an individual’s outcome expectancies, and their psychological resources. Transactions are mediated by a process of perception, cognitive evaluation (i.e., appraisal), and coping, which in turn results in positive or negative emotional and behavioral responses, and affects well-being outcomes (see Lazarus, 1999). Moreover, this ongoing process is moderated by a range of personal (e.g., trait anxiety) and situational (e.g., social support) characteristics (Fletcher and Scott, 2010; Olusoga et al., 2012). While a number of recent investigations have examined the stressors encountered by coaches (e.g., Potts et al., 2018), coaches’ appraisals and coping (e.g., Didymus, 2017), and the implications of these on coaches’ well-being (e.g., Norris et al., 2017), little attention has been devoted to understanding the role of the personal and situational characteristics that moderate the stress process (Knight et al., 2013). One such personal characteristic, which may distinguish between those who do and do not cope well in demanding situations, is *hardiness* (Fletcher and Scott, 2010; Mazerolle et al., 2018; Bartone and Homish, 2020).

Hardiness is defined as a multidimensional form of dispositional resilience^1^, and is associated with improved mental health and performance under stressful conditions (Kobasa, 1979; Bartone and Homish, 2020). Central to this disposition are three interrelated attitudes: *commitment* (i.e., the ability to persist with effort, even when experiencing a range of stressors); *control* (i.e., the ability to feel and act as if one is influential in the face of the varied contingencies); and *challenge* (i.e., the belief that change is normal in life and that change is an incentive for growth; Kobasa, 1979). Specifically, in response to potentially stressful situations, hardy individuals display an internal motivation and *commitment* to the various areas of life, including work, interpersonal relations and self, a greater belief in their own ability to *control* or influence the course of events, and an appreciation of new experiences and *challenges* as opportunities for learning (Stein and Bartone, 2020). Consequently, hardy individuals are courageous when encountering new experiences and handling disappointments, tend to be highly competent, and while not impervious to the negative effects of stress, are resilient in responding to highly demanding situations (Bartone, 2012; Mazerolle et al., 2018).

An extensive body of empirical research, across a range of demanding environments and occupations (e.g., military, Maddi et al., 2012; firefighting, Maddi et al., 2007), has demonstrated that hardiness may buffer the ill-effects of stress on health and performance. Within the context of sports, researchers have shown that athletes higher in hardiness report more facilitative interpretations of anxiety (e.g., Hanton et al., 2013), experience fewer sports injuries (e.g., Wadey et al., 2012), and demonstrate greater stress-related growth following injury (e.g., Salim et al., 2016). With respect to coaches, high-hardy coaches appear less susceptible to burnout due to: (a) reduced perceptions of the significance of environmental demands (e.g., Kelley et al., 1999; Hendrix et al., 2000); and (b) the experience of lower levels of work–life conflict (Mazerolle et al., 2018). When combined with the findings from other demanding environments and occupations, there is now a considerable amount of support that hardiness is an important personal characteristic that protects individuals against the potentially negative effects of stress (Fletcher and Scott, 2010). A possible explanation in relation to Lazarus and Folkman’s (1984) transactional stress theory is that hardy individuals have increased perceptions of control over a stressor, are more likely to make challenge appraisals (e.g., see potential growth from a demand) and are willing to commit to more effective approach-based coping strategies (Hanton et al., 2013).

Despite the potential for hardiness to protect individuals against the stressors they encounter, the development of hardiness remains a topic of much debate. For some, hardiness represents a stable, trait-like personality disposition that falls within the general trait theory of personality (cf. Matthews et al., 2003). For others, although hardiness develops early in life and is reasonably stable over time and across situations, it is not immutable and can be continually shaped throughout one’s lifespan (Bartone and Barry, 2011). Indeed, a number of programs have been developed to train hardiness, with some success (Bartone et al., 2016; Stein and Bartone, 2020). Beyond such training programs specifically designed to develop hardiness, however, little is known about how it is shaped by experience and social context (Hystad et al., 2015). Addressing this gap in the research and increasing our understanding of how hardy dispositions are cultivated could make a significant contribution to the sport and related performance psychology literature. Specifically, within a sport coaching context, such research would help to further explore the coach as both a performer and as a person (cf. Cropley et al., 2020b). In doing so, findings could support the development of an evidence-base that has the potential to inform both applied sport psychology practice and coach education programs aimed at facilitating coaches’ abilities to cope, and even thrive, with the ever-increasing demands of elite sport.

The purpose of this project, therefore, was to address the dearth of research that has explored the development of hardy dispositions and examine the cultivation of hardiness in sports coaches. In order to achieve this goal, we conducted a systematic multi-study project. In Study 1 (qualitative phase) we examined pre-determined elite, high-hardy coaches’ perceptions of how they developed their hardy beliefs over time. In Study 2 (quantitative phase), we examined key findings from Study 1 regarding the relationship between developmental factors and augmented hardiness with a large sample of coaches, with the view to quantify the magnitude of these associations. Thus, we sought to improve understanding of how we might better train and support coaches in developing hardy beliefs to protect them from the increasing demands associated with the coaching role. In doing so, we aimed to make a novel and significant contribution to current knowledge in the area.

## Research Design

The research we present in this article (Studies 1 and 2) is underpinned by the perspective of *critical realism* (Bhaskar, 1975; Lincoln et al., 2011). Critical realism is a philosophical position in which researchers perceive the existence of an objective world but recognize that knowledge is a subjective, discursively bound (i.e., transitive) social construction (Edwards et al., 2014). While this position has been questioned due to the tension it causes concerning the definition of ontology (see Cruickshank, 2004), critical realists reject the single dichotomy perspective (e.g., between positivism and constructivism) due to the epistemic fallacy that is thought to exist (Danermark et al., 2002). Here, it is proposed that the tendency to couple ontology and epistemology confuses that which exists and what is known about it, and thus, critical realists perceive a *middle ground* (North, 2017). Therefore, epistemologically, knowledge is determined by the problems we are faced with and the questions we ask about the world around us (Danermark et al., 2002). For critical realists, this knowledge is developed through observations in relation to understanding causal mechanisms (e.g., that certain developmental mechanisms will influence an individual’s level of hardiness and that these mechanisms will be individually interpreted). Consequently, we have adopted an *exploratory sequential mixed-method design* in which a qualitative component (e.g., Study 1) is used to generate new conceptual knowledge followed by a quantitative component (e.g., Study 2), which is used to investigate and test that emerging theory (see Hesse-Biber, 2014; Anguera et al., 2018). This approach was deemed most appropriate given our aim and the limited understanding regarding how sports coaches might develop hardy dispositions, the potential impact of hardiness on coach functioning and success, and the causal strength between developmental mechanisms and augmented levels of hardiness.

## Study 1: Elite Sport Coaches’ Perceptions on Hardiness Development

### Research Aims

The aim of this study was to explore how pre-determined elite, high-hardy coaches developed their hardy dispositions. Specifically, using a critical incident approach (Hanton et al., 2009), we interviewed coaches to explore how certain critical experiences inside and outside of their sporting lives helped them to develop hardiness and the attitudinal sub-components of control, commitment, and challenge. Alongside this primary aim, we also explored how being hardy impacted on their functioning and success (e.g., personal performance outcomes) as a coach.

### Materials and Methods

#### Sample Selection and Participants

In order to ensure that the participants sampled would be best placed to discuss the development and impact of hardy dispositions, purposive criterion sampling was employed (Patton, 2015). First, given that recent research has highlighted the multitude, complexity, and significance of the demands experienced by *elite* coaches, requiring them to exhibit the personal characteristics associated with effective and adaptive coping (e.g., Olusoga et al., 2012; Didymus, 2017), an elite sample was selected. Elite coaches were defined as those working at an international level or professionally at the highest national level of their sport (cf. Swann et al., 2015). Second, participants were required to have been coaching at the elite level at the time of the study to ensure that current insights could be explored. Finally, participants were only selected if they were deemed to be high in hardiness. To determine this, all prospective participants were asked to complete the Dispositional Resilience Scale (DRS, Bartone et al., 1989), which measures each of the three sub-components of hardiness (control, challenge, and commitment), providing individual component scores and an overall hardiness score. The measure consists of 45 items (15 for each sub-component) rated on a 4-point Likert scale [0 (not at all true) to 3 (completely true)], resulting in hardiness scores of between 0–135. The DRS has been shown to have high levels of internal consistency and sufficient levels of convergent validity, with Cronbach alpha coefficients for hardiness, control, challenge and commitment being reported within a sporting sample to be 0.84, 0.72, 0.71, and 0.70 respectively (Hanton et al., 2013). Using a quintile split to rank potential participants (see Hassler, 2018), only those who scored 108–135 on the DRS were classified as high hardy.

Twenty five potential participants (male = 15; female = 10) were initially contacted through the National Governing Bodies of their respective sports and invited to participate in the pre-screening procedures to check whether they met the sampling criteria. Participants were asked to complete an electronic version of the DRS via an online link, which also contained ethical information about the nature of the study, their rights, and a request to confirm their voluntary consent to participate (all of whom did). Subsequently, only those who scored in the highest DRS quintile were selected to participate in the study itself. The final sample (*n* = 13) consisted of five senior national (e.g., English Premier League) and eight international team sport coaches (male = 9; female = 4; *M*_*age*_ = 40.3 years; *SD* = 6.7), who collectively worked across a range of sports (e.g., netball, football, rugby league, triathlon). Participants were all classified as high hardy [DRS scores ranging from 108 to 130 (*M* = 117.1; *SD* = 7.87)], had been coaching for an average of 8.69 years (*SD* = 4.1), and had been in their current position for an average of 2.7 years (*SD* = 1.3) at the time of the study.

#### Data Collection: Interview Guide

Due to the exploratory nature of this study, a semi-structured interview approach was adopted (cf. Patton, 2015; Potts et al., 2018). Aligned to our philosophical position, and the notion that hardiness is situated within the context of adverse experiences (cf. Stein and Bartone, 2020), the interview was underpinned by a critical incident approach (see Hanton et al., 2009). Specifically, Hanton and colleagues suggested that such an approach encourages a level of reflection that elicits deeper and more meaningful insights into participants’ experiences. As a result, we asked participants to discuss the three most influential experiences they had faced across their lifespan that had helped them to develop their hardy dispositions. The critical incident approach allowed us to frame their development more widely and explore the processes underpinning the incident that resulted in positive adaptations to hardiness and its sub-components (i.e., challenge, control, commitment).

Following introductory statements regarding the nature of the study and a reminder of the participants’ rights, the interview guide consisted of four main sections. First, a series of preliminary questions, focusing on exploring the participants’ thoughts concerning the importance of the psychology of the coach, were used to settle the participant into the interview and to frame the subsequent discussions. Second, participants were guided through a detailed examination of each of their critical incidents (e.g., “Please explain the nature of the incident in as much detail as possible”; “What did you learn?”; “How did this influence the level of your hardiness?”; “How does this incident relate to the wider situations you have experienced that influenced your hardiness?”). Third, participants were asked to discuss how their hardy dispositions had impacted on their coaching practice and success. Finally, participants were offered the opportunity to add any further information in relation to their experiences before the interview was concluded with a series of questions concerning the nature of the interview itself (e.g., “How do you feel the interview went?”; “Were you able to tell your full story?”).

A pilot interview was conducted with a sub-elite, high-hardy, female football (soccer) coach to assess the efficacy of the guide in relation to the study aims and its suitability for facilitating in-depth reflection. Following participant feedback, the interviewer’s reflections and research team discussions, minor modifications were made to the phrasing of questions and additional neutral, non-directional probes were prepared (e.g., “What do you mean by this?”).

#### Procedure

Participants who met the sampling criteria were contacted via email to inform them of the nature of the interview, their rights as participants, the request for voluntary written consent, and subsequently to arrange a suitable time and location for the interview to take place. Once consent had been received and the logistics agreed, each participant was sent an interview preparation booklet, which was designed to: (a) further their understanding of the purpose of the study; (b) prepare them for the nature of the interview by offering definitions of hardiness and its sub-components; and (c) encourage them to consider those incidents that they deemed as most critical in the development of their hardiness so as to aid recall during the interview itself (cf. Hanton et al., 2009; Patton, 2015). All interviews were conducted face-to-face by the second author in a neutral location (e.g., hotel meeting room) selected by the participant to make them feel comfortable and aid the flow of the conversation. Interviews lasted between 45 and 98 min (*M* = 74; *SD* = 16.7), were audio-recorded in their entirety, and were subsequently transcribed verbatim.

#### Data Analysis and Methodological Rigor

In accord with our critical realist perspective, the general aim of Study 1 was to generate knowledge (e.g., how hardiness might be developed) through a process of inductive inquiry. Subsequently, the mechanisms and underlying structures of this emergent knowledge could then be investigated (Study 2) to offer a more thorough examination of factors that may augment hardiness (Hu, 2018). Following procedures advocated by Braun and Clarke (2012), data were, therefore, analyzed thematically using a six-step inductive approach.

First, transcripts were read and re-read to ensure familiarity by all authors. Second, authors two and five independently conducted initial coding to identify meaningful ideas within the data related to the research aims (e.g., the impact of *work-related demands* on hardiness development). Following this, comparative analysis and discussion took place between authors two and five, with author one acting as a critical friend to question any potential bias in the initial coding and facilitate discussion to settle on the final codes (Smith and McGannon, 2018). Third, authors two and five collectively organized codes that shared similar semantic qualities into descriptive themes (i.e., second order themes). For example, social, personal, and work relationships that facilitated coping were organized into the descriptive theme of *social support*. Fourth, the same authors interpreted the relationship between the descriptive themes they both identified. This helped to interpret the meaning of the descriptive themes and resulted in the development of overarching interpretive themes (i.e., third order themes) that could be presented in the form of a hierarchical network. Fifth, to address the rigor of the analysis, authors two and five critically discussed the definition of each theme to ensure that it was clear, distinct, and traceable back to the raw data (Didymus, 2017). Finally, the themes were presented to the entire research team, who, acting in the role of critical friends, encouraged reflection on the data, the actively created themes, their definitions and the process of analysis. This process allowed the researchers to justify interpretations of the data and thus improve confidence in the process and outcomes of the analysis (Smith and McGannon, 2018).

A range of strategies were also adopted to enhance methodological rigor including: (a) meeting appropriate ethical standards; (b) selecting an appropriate, information rich (e.g., high-hardy) sample; (c) testing the interview guide (e.g., pilot study); (d) preparing participants by ensuring a clear understanding of hardiness so that this construct could be discussed, rather than potentially discussing related psychological factors; and (e) providing participants the opportunity to comment on the rigor of the interview process (cf. Tracy, 2010).

### Results

**FIGURE 1:**
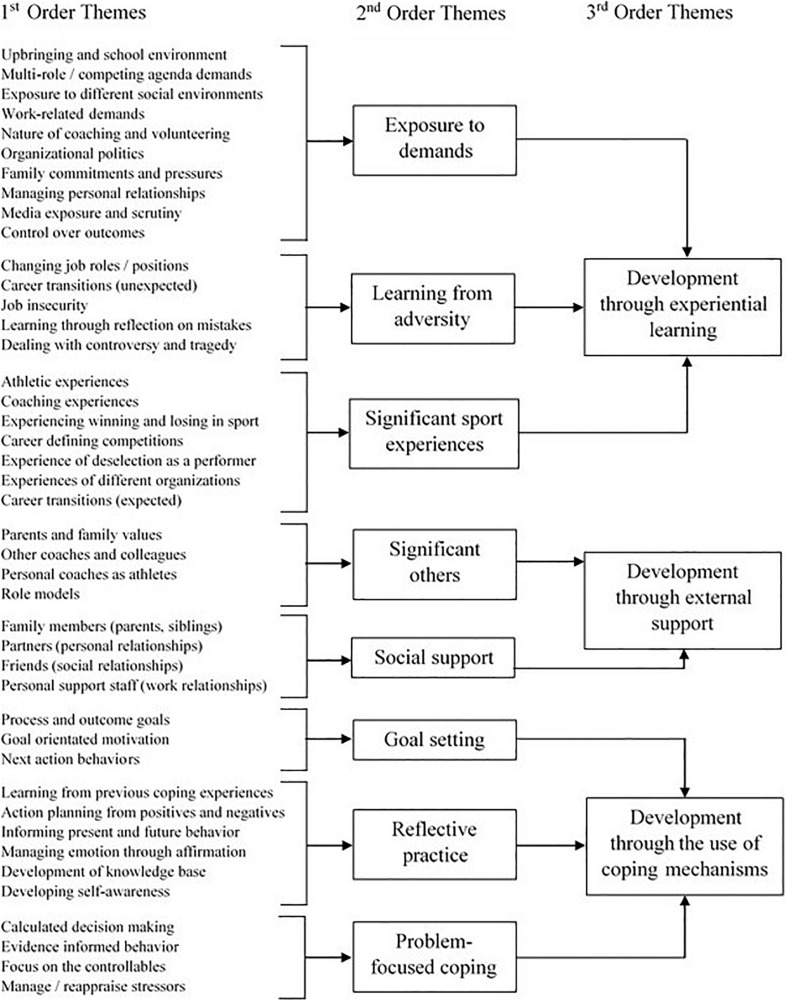
Hierarchical analysis of hardiness development themes.

Through the data analysis procedures we identified three higher order themes relating to the development of participants’ hardiness: (a) development through experiential learning; (b) development through external support; and (c) development through the use of coping mechanisms. These, and their lower-order themes, are presented as a hierarchical network (Figure 1). This network is accompanied by descriptive narratives that center on the development of hardiness. Alongside this developmental focus, the impact of hardy dispositions on participant functioning and success is presented in relation to each of the higher-order themes and their corresponding lower-order themes. Within each narrative, a selection of representative participant quotes are provided to offer insights into the raw data and the participants’ experiences (Patton, 2015).

#### Development Through Experiential Learning

##### Exposure to demands

The importance of being exposed to a wide variety of demands, across the different areas of the participants’ lives, for developing hardy dispositions was reported by all participants. Specifically, participants referred to the learning opportunities that such exposure provided them to better understand how they controlled, managed, and/or coped with those demands. For example, one participant stated, “Although the contexts are different, I learned early that all aspects of work and life throw you so many difficult challenges. I knew that if I could cope with them I would be able to cope with anything in sport.” Another indicated:

I was studying for a masters; I had about 37 hours of work as a coach and volunteering. I was coaching football at xxx (club), I was also coaching at two football development centers and I was still an elite athlete at that point … being challenged in different ways really helped me to be more robust when I was faced with challenges later on.

Other participants referred to specific demands they had experienced that had resulted in a process of experiential learning and thus led to positive adaptations to their hardiness. One participant outlined, “Once you’ve experienced that prickly interview … after a bad game, and you’ve still delivered a good message, you quickly learn to see the media as a test – an opportunity to present yourself as in control.” Similarly, another participant detailed, “Learning to manage with the breakdown of a relationship (divorce) really teaches you how to manage some of the nonsense that happens above you in an organization and the relationships you have with those people (board members).” Such statements represent many of those presented by all participants regarding the necessity of experiencing difficult situations in different contexts for the development of their hardy beliefs.

When asked how the exposure to numerous demands contributed to the development of hardiness, one participant suggested, “… you learn to see the issue differently … you learn to see it as a challenge and you make decisions about how you meet the challenge head on, in a positive way.” Other participants indicated, “You begin to understand that you can’t control everything so you need to put your effort and energy into the things that you’ve got some control over” and “It (critical incident) showed me that I can handle a lot of work and stress and I won’t break down irrespective of the pressure. On reflection, it showed me that I can achieve success and balance and spin plates.” Consequently, it was generally understood that the participants’ exposure to demands was a worthwhile process because of the resultant learning that took place. Thus, the process of experiential learning appeared to influence the participant’s perceptions of the nature of, and control over, future demands, and was therefore noted as being a key contributor to developing hardiness.

##### Learning from adversity

In addition to being exposed to a range of demands, the participants outlined how learning from adversity had also helped to enhance their hardiness, particularly through the development of its associated attitudinal components. For example, participants suggested, “That situation (adversity) taught me that you can’t predict what’s going to happen to you so you have to focus on the positive things you can do to move forward and commit to them – don’t take a step backwards” and “What I felt at that time wasn’t good, but I still worked – still did what I could. I think I was really brave when I eventually held my hands up and asked for help … that bravery has stuck with me.”

Participants detailed experiencing a number of adversities, including: *dealing with controversy*, *unexpected career transitions*, *losing their jobs*, and *dealing with personal tragedy*. For example, participants stated, “I wouldn’t say I’ve experienced many of them (adversities), but getting the sack from xxx (club) was particularly tough. “It taught me to enjoy every moment … to stay focused and stay true to yourself” and “When my dad died I struggled … having time to reflect, it reminded me that he’d taught me so much about everything, so I knew I could cope and move forward. Strangely it probably took that for me to see.” Another participant reported:

On my first day at school, this kid says to me, “Do you want to fight?” … I look back and it was awful but it shaped who I am. I’ve had to battle for everything, whether it was getting into the team or simply surviving the playground.

Consequently, the relatively novel nature of the adversity the participants experienced provided unique opportunities to learn about themselves and their personal resources that helped them to manage those situations and, in some instances, demonstrate positive adaptations.

##### Significant sport experiences

Throughout all of the interviews, each participant explained that, while they had learned from situations experienced in all areas of their lives, significant experiences within sport were a key contributor to the development of their high levels of hardiness. Participants indicated that these experiences had, “Shaped who I am as a coach,” “Made me become more resilient,” and “Helped me to understand how to be tough – how to enjoy the challenge.” One participant explained, “I guess the learning from that experience (critical sporting incident) is really taking stock of the finer details … you’ve got to back yourself and show to the group you work with that you are really are in control.”

Participants were clear that learning about the context of sport and performance allowed them to understand how to function effectively, despite the nature of the organizational and environmental conditions in which sport takes place. As a result, some participants felt as though their experiences in sport allowed them to understand the importance of being hardy and how to be successful. For example, “Winning xxx (tournament) took a lot of focus and effort. It’s in those moments, when you think back, that you understand how resilient you are, or hardy as you put it … you don’t get that from other situations” and “I made errors as a player that cost us games, but I bounced-back. At the time you don’t think about it much but when I started coaching I understood what I could do and that’s helped me in this role.”

#### Development Through External Support

##### Significant others

Significant others were identified by participants as those people who had played a key role in their personal and professional development. Eleven of the coaches within this study suggested that significant others (e.g., other coaches, colleagues, parents) contributed to shaping their knowledge, attitudes, and behaviors during their everyday lives and careers. For example, “My parents did everything possible to give me a chance (in sport). Looking back, it’s humbling really. I think their selflessness, drive and work ethic certainly rubbed off on me. Trying to make them proud has definitely focused me” and “I became really close with xxx (coach). We talked about the things she’d been through and still managed to be successful. That helped me to understand that I could do the same – that I had the mental attributes to thrive.” In developing hardiness, the examples provided by participants indicated how social approaches to learning and development were considered as essential. Indeed, participants implied that such interactions influenced their outlook on life and sport and helped them to gain perspective within their roles, which helped them to function effectively. In summary, “Those personal and professional relationships help you to understand who you are, your strengths and weaknesses … but they also help to shape the way you see things, the way you put the challenges you’re faced with into perspective.” Some participants also outlined how these significant others did not necessarily provide positive influence through their behaviors and attitudes, but such behaviors helped the participants shape their own coaching style. For example, one participant stated, “Having had the coaches that I had … taking all of the worst bits and making sure I wasn’t like that was important … I suppose it gave me a sense of understanding of negative coach behaviors.”

##### Social support

Social support differed from *significant others* in that the support from significant others was generally observed, whereas social support represented the explicit and direct social interactions with others that helped participants cope with, and manage, the demands associated with their professional roles. In doing so, such social support was deemed to have had a particular impact on the development of participants’ hardiness. For example, participants reported, “My parents gave me unconditional support … when those (media) reports were published they were there reminding me of my achievements and stopped me feeling sorry for myself. I suppose that support helped me stay in control of the situation” and “Having someone there to act as a sounding board has been important for me. It’s helped to frame the best approaches to deal with issues and to check in with someone to ensure that you’re giving everything to the cause.” In light of these examples, participants were clear about the contribution that social support played in supporting adaptations to their beliefs concerning control, challenge, and commitment. Specifically, participants indicated that social support helped to alter their perceptions and interpretations of the demands that they experienced and instill an approach to proactively deal with such demands. This sentiment is best summarized by one participant who stated, “Getting honest feedback from those you trust is key because you see the problem differently. It’s no longer something you can’t control but something that you can manage, that will make you better. That’s an attitude you need.”

#### Development Through the Use of Coping Mechanisms

Participants discussed using a number of specific mechanisms throughout their lives that they believed had not simply allowed them to manage stressors, but instead had been transformational in nature (i.e., approach-based coping designed to actively problem solve or facilitate growth), and thus had positively impacted their hardiness.

##### Goal setting

One strategy reported by most participants was that of goal setting, which allowed for the participants’ strength of commitment to flourish, “I had specific end goals in my playing career and in order to achieve those goals no one aspect could derail me … Yes, I think that commitment needed to achieve a goal really builds your hardiness.” Another participant reported how goal setting helped instill a sense of control over, and commitment to, the situation: “Giving myself specific targets really helps me to apply a level of control over my work. They also help to ensure that I stay focused and committed to positive action.” Similarly, the strategy of goal setting also appeared to provide a sense of clarity and focus that influenced the resilient disposition of hardiness: “I knew I always wanted to coach xxx (national team) and I knew what I needed to do. I set targets for my development and I dedicated myself to getting those targets. Staying focused, that’s what makes you tough.” Linked to this, a number of participants discussed the importance of “next action behaviors” in moving on from negative situations (e.g., losses) to focus on factors that they could control, “When you’re under the cosh as a team, and as a coach, you can easily get lost. I just set myself little challenges, little goals to keep me moving forward, to stop me from dwelling on the past.”

##### Reflective practice

All participants reported the use of both systematic and informal approaches to reflective practice (RP) after demanding events in order to learn and inform future behavior. One participant stated:

You experience a situation; you reflect on it and that reflection helps you deal with similar situations in the future. I reflect on everything, I don’t want to go into the theory of the reflective cycle but I think when you do something, you reflect on what went well, what didn’t go so well and how I might make those changes. So, for me reflection is a fundamental part of my coaching and it’s really helped me to be hardier personally.

RP was identified as a coping strategy due to the process being associated with “knowledge gains” that helped coaches to deal and cope with the different demands they experienced in their lives and, as a result, instill a great sense of control, commitment, and challenge. For example, “Reflection’s really helped me to understand myself, to become more aware of what works for me and how then I can be positive in difficult situations” and “Using reflective practice has really given me a different perspective on things. It helps me to stay rational, understand what needs to happen, set targets and engage in the right behaviors to achieve those” and “Without reflection I wouldn’t have learned (critical incident); it’s the process that draws things together, helps you to realize what you need to do to succeed and, therefore, gives you confidence to take action.” RP was thought to help participants also become more familiar with demanding situations and, as a result, feel more prepared to be proactive in their approaches. Specifically, “(Because of reflection) I know what’s coming up … because of learning about those previous instances before they happen and I like to think I’m pretty tooled-up when they happen.” Other participants acknowledged how engaging in RP emanated from their commitment to improve. For example, “You have to be committed to reflect all the time, on everything, but that comes from that inner drive to improve. I want to get better every day, every experience and so reflection’s a huge part of that journey.”

##### Problem-focused coping

Throughout the discussions with all participants a number of problem-focused approaches to managing demands were discussed, including: “placing focus onto those factors that could be controlled”; “seeing the issue differently, as a positive challenge” (e.g., reconstructing the way in which the problem is viewed to direct approach-based behaviors); and “making calculated, informed decisions rather than reacting rashly.” Specifically, one participant stated:

Having 90–95% control and only having to react to 10% will definitely help you as a coach. Having structures and a contingency plan, dealing with the “what if” scenarios, so when they happen in the game you have anticipated them and are prepared.

These approaches were reported to influence the perception of control that the participants had in different situations and, therefore, helped them to feel better prepared for the difficulties they encountered. One participant reported, “When xxx (family member) passed away I couldn’t control that; there was nothing I could do, but focusing on what I could do to support my mum and the rest of my family helped to make sure I acted as positively as I could.” Participants detailed that many of these approaches and attitudes had developed over time through learning from their experiences rather than through formal education. For example, “I flew off the handle with (player), it made me feel better at the time but it didn’t really help. Reflecting back, I knew I had to be more managed, controlled … you don’t get taught that” and “I’m pretty good at seeing things for what they are rather than through emotion that distorts them. I think that’s something I’ve developed over time, through experience of tough situations.”

### Study 1: Key Findings and Summary

Given the considerable demands associated with sport coaching, there have been calls for researchers to explore the personal characteristics that may buffer coaches’ experience of stress (Olusoga and Thelwell, 2017; Cropley et al., 2020b). One such personal characteristic, widely highlighted as a mechanism that protects individuals from negative responses, is *hardiness* (Stein and Bartone, 2020). Little research has, however, explored how hardiness might be developed. The current study attempted to address this gap by interviewing elite, hardy coaches about their experiences of developing hardiness. In support of early conceptualizations of hardiness (e.g., Kobasa, 1979) and more recent sport literature that has considered this personal characteristic (e.g., Mazerolle et al., 2018), we have shown that the development of hardy dispositions helped coaches to: (a) view situations as positive challenges and opportunities for growth; (b) engage in a range of strategies to exert control during difficult circumstances; (c) commit to proactive approaches to managing their demanding lives; and (d) embrace adaptive attitudes toward coping with stressors. Hardiness was, therefore, attributed to the participants’ ability to manage personal and professional demands while demonstrating the adaptive behaviors that helped them to thrive and be successful.

From a developmental perspective, this study identified a number of important personal (e.g., coping mechanisms) and situational (e.g., exposure to demands and adversity) factors thought to contribute to increases in hardiness. Of particular relevance was how participants engaged in a process of experiential learning following demanding situations, which resulted in a higher propensity to manage future stressful episodes. These findings offer novel empirical support for recent literature in which researchers have argued for the potentially adaptive role of stress (e.g., Crane et al., 2019). Indeed, Seery and Quinton (2016) reported that stressors may have a resilience-strengthening effect, with the findings of our research providing a similar contention for the disposition of hardiness. That is, engaging with stressors may have positive consequences for the long-term development of hardiness and its sub-components (i.e., control, challenge, and commitment). Our findings also provide insight into how different experiences and coping mechanisms relate specifically to the different attitudinal sub-components (e.g., familiarity with the sporting context linked with control; exposure to demands and adversity linked with challenge). Hardiness is widely conceptualized as a form of dispositional resilience, and while it is developmental in nature the process of augmenting the construct can be difficult. Hystad et al. (2015), for example, found little change in military personnel hardiness following a 3-year, longitudinal training program designed (in part) to augment the construct. Consequently, in accord with our findings, we posit that practitioners may be better served by targeting the development of each individual sub-component to facilitate overall higher levels of hardiness. Indeed, research has identified individuals who demonstrate unbalanced hardiness profiles (e.g., high control and commitment, low challenge), suggesting that while the sub-components correlate to produce hardiness they may also be formed independently (Johnsen et al., 2014).

Perhaps fundamental to the participants’ hardiness development in this study was their engagement in RP as a process to facilitate meaningful learning from their experiences. Participants recognized RP as both a coping strategy and as the key process in supporting greater self-awareness and insight (e.g., understanding of thoughts and feelings), both of which are thought to be intrinsically linked with the sub-components of hardiness (Cowden and Meyer-Weitz, 2016). Participants also detailed how reflection enabled them to transform their experiences into learning, which generally elicited adaptive actions within future demanding situations and facilitated the development of coping mechanisms that supported their hardy dispositions. Indeed, it has previously been argued that learning is not simply a natural consequence of having an experience (e.g., Hanton et al., 2009). Instead, individuals must seek to reform their experiences through meaningful reflection in order to develop increasingly adaptive beliefs and the capacity to effectively implement coping and emotion regulation repertoires (Crane et al., 2019). Certainly, RP, which is viewed as a positive approach to learning from experience and future action, is widely considered as a transformative process that facilitates the development of the tacit knowledge individuals need to: (a) improve congruence between beliefs, values, and behaviors; (b) increase self-awareness; (c) enhance the effectiveness of action; and (d) improve their ability to think rationally and problem-solve (see Cropley et al., 2018).

Importantly, the participants in the current study demonstrated a commitment to RP in which they largely considered their engagement in the process as systematic and deliberate. Historically, it has been argued that RP can occur at different levels of insight, from technical (i.e., performance reviews) to critical (i.e., challenging habitual practice; Knowles et al., 2012). Many authors have proposed, however, that transformative outcomes are more aligned to critical levels of RP as a result of the deeper inter- and intra-personal examination that takes place at that level (Cropley et al., 2018). It would appear, therefore, that while RP can potentially elicit the self-awareness and transformational adaptations required to enhance levels of hardiness, individuals need to be committed and skilled reflective practitioners to achieve such outcomes. Developing an individual’s ability to engage in critical levels of RP may consequently offer practitioners an alternative mode of intervention that supports a coach’s aptitude for responding positively to demands (Crane et al., 2019).

In this study, we have shown that RP is a potentially vital mechanism for facilitating hardiness and associated behaviors. Some support for this contention has been provided by Cowden and Meyer-Weitz (2016) who previously found positive correlations between reflection, self-awareness, and resilience in competitive tennis players. However, Cowden and Meyer-Weitz’s research focused specifically on resilience rather than the disposition of hardiness and did not explore participants’ reflective abilities – only that they actually engaged in RP. Consequently, the links between an individual’s propensity to engage in RP, their skill in being able to reflect at critical levels of insight, and their levels of hardiness are still somewhat intuitive. In spite of the findings of our current study, and recent literature in which authors have proposed that reflection on stressors can enhance individuals’ resilience to adversity (e.g., Crane et al., 2019), little is known about the relationship between hardiness and RP. For example, the current research was conducted with elite coaches identified as high hardy, so it is unclear whether RP facilitates higher levels of hardiness and associated beliefs in the wider population of sub- and non-elite coaches. As a result, we conducted a follow-up study to explore the potential relationships between hardiness and an individual’s propensity and ability to engage in RP.

## Study 2: Coach Reflective Practice Profiles and Differences in Hardiness

### Research Aims

This study aimed to identify profiles of RP tendencies among sport coaches and investigate the differences between these profiles on levels of hardiness. To strengthen our understanding of potential relationships, we adopted an exploratory design and a person-centered analysis. Specifically, we aimed to cluster participants based on their RP profiles (e.g., participants’ level of engagement in reflective practice, and the level of their ability to reflect) allowing us to examine how different profiles may influence each of the sub-components of hardiness.

### Materials and Methods

#### Participants

Participants were recruited through National Governing Bodies, social media, and snowball sampling procedures under the premise that they were in a coaching role at the time of data collection. The final sample consisted of 402 (males = 320, females = 80, not identified = 2) multi-national [United Kingdom = 222; United States = 142; Australia = 19; other = 19 (full breakdown available on request)], team (*n* = 355) and individual (*n* = 47) sport coaches. The participants worked at the youth (*n* = 228), senior (*n* = 92), or both youth and senior (*n* = 82) levels of their respective sports, and covered a range of performance levels, including: grassroots (*n* = 34); local/regional clubs (*n* = 149); national clubs (*n* = 109); national representative teams (*n* = 36); international (*n* = 44); and other (*n* = 30). The participants ranged in age from 18 to 68 years (*M* = 37.7; *SD* = 12.6) and in coaching experience from 1 to 28 years (*M* = 11.6; *SD* = 7.1).

#### Measures

##### Hardiness

To assess hardiness, we used the Dispositional Resiliency Scale-15 (DRS-15; Bartone, 1995), which is a shortened version of the 45-item scale adopted in the first study of this article. This is because the shortened version has: (a) the advantages of brevity (given the nature of data collection and of the sample, this was deemed important); and (b) improved psychometric properties (cf. Hystad et al., 2015). The DRS-15 includes positively and negatively keyed items “about life in general that people often feel differently about” and measures hardiness and its sub-components (i.e., commitment, control, and challenge; five items per sub-component). Participants are asked to indicate the truthfulness of each statement for them on a 4-point Likert scale anchored at 0 (not at all true) and 3 (completely true), with scores for each subcomponent ranging from 0 to 15 and the composite hardiness score from 0 to 45. The DRS-15 has demonstrated acceptable test-retest reliability (0.82), with corresponding test-retest coefficients for the sub-components being reported as: commitment = 0.76; control = 0.76; and challenge = 0.72 (Dixon and Bares, 2018).

##### Engagement and need for reflection

Participants’ engagement in reflection and their level of self-awareness was assessed using the Self-Reflection and Insight Scale (SRIS; Grant et al., 2002). This measure consists of two sub-scales: *self-reflection* (SRIS-SR) and *insight* (SRIS-IN). Self-reflection is also split into two related components, *engagement in self-reflection* (SRIS-SRE) and *need for self-reflection* (SRIS-SRN), and measures the recognition of the need for and the process of engaging in reflection. Insight assesses the “clarity of understanding one’s thoughts, feelings, and behaviors” (Grant et al., 2002, p. 821). The SRIS contains 20 items, with 12 items measuring SRIS-SR (6 items each for SRIS-SRE and SRIS-SRN) and 8 items for SRIS-IN. Each item is rated on a 5-point Likert scale (1 = strongly disagree to 5 = strongly agree). Nine items are reverse scored before summation to obtain subscale scores (Cowden and Meyer-Weitz, 2016). Convergent and construct validity for the SRIS have been evidenced, along with good 7-week test–retest reliability (SRIS-SR, 0.77; SRIS-IN, 0.78) and strong Cronbach’s alphas (SRIS-SR, 0.91; SRIS-IN, 0.87) for the subscales (Grant et al., 2002).

##### Level of reflection

To assess the level (and associated quality) of participants’ RP, the Questionnaire for Reflective Thinking (QRT; Kember et al., 2000) was adopted. In line with Mezirow’s (1981) theory of transformative learning, the QRT contains four sub-scales (16 items, 4 items per sub-scale), which collectively assess the extent to which individuals are able to engage in reflective thinking. Two sub-scales, *habitual action* (e.g., activity taken automatically with little or no deliberate thought that is based on and is a result of previous learning) and *understanding* (e.g., thoughtful action that makes use of existing knowledge, without attempting to appraise that knowledge) assess non-reflective actions. Two sub-scales, *reflection* (e.g., the critique of assumptions about the content/process of problem solving) and *critical reflection* (e.g., the testing of premises to transform meaning and action) assess reflective actions. The QRT utilizes a 5-point Likert response scale (1 = definitely disagree to 5 = definitely agree), with sub-scale scores ranging from 4 to 20. The QRT has acceptable internal consistency (0.63–0.76 Cronbach’s alpha) and construct validity has been supported through confirmatory factor analysis (Lethbridge et al., 2013).

#### Procedure

All three measures (and instructions for completion) were uploaded to Online Surveys together with participant information, consent details, and a request for demographic information (e.g., age, sport-type). From this, an electronic link (accessible on all forms of electronic media) was created to allow the questionnaire pack to be widely distributed as well as provide potential participants the opportunity to take part at a time and location of their choosing. A number of National Governing Bodies agreed to distribute the link to their coach networks, and a request was made for participants across a range of social media platforms. Potential participants were instructed that they needed to complete the entire pack in one sitting, but that the link would remain open for a period of 2 months. Given that all questions were made compulsory, participants not wishing to answer a question or complete the entire battery of measures were informed that they could cease their participation at any time. As a result, only complete cases were recorded and stored on Online Surveys, meaning that there was no missing data.

#### Data Analyses and Rigor

We executed a two-step analytical approach that reflects contemporary recommendations for mixture modeling (Asparouhov and Muthén, 2018; Morin et al., 2020). In the first step, we identified profiles of participants who share commonalities in their reflection scores using latent profile analysis (LPA) with a robust maximum likelihood estimator (MLR). Briefly, LPA involves probabilistic model-based clustering to identify homogenous subpopulations of cases according to unique configurations of scores on several continuous indicators (Wang and Hanges, 2011). In so doing, LPA incorporates measurement error directly into the statistical model and quantifies via posterior probabilities the likelihood that cases belong to one profile rather than the other profiles. We used factor scores of seven latent *reflection* variables obtained from a correlated seven-factor measurement model as indicators of the LPA to control partially for measurement error (i.e., more weight to items that are most reliable; Skrondal and Laake, 2001) and maximize interpretability via standardization with a mean of zero and variance of one (Morin et al., 2020). We calculated McDonald’s Omega (ω) as an estimate of internal reliability using Hayes and Coutts’s (2020) SPSS macro. Intercepts and variances of profile indicators were freely estimated to maximize congruence with a realistic characterization of real-world phenomena and generation of accurate parameter estimates (Morin et al., 2011; Peugh and Fan, 2013). We estimated models including one to eight profiles to determine the structure that best represented a balance between model fit and parsimony (Nylund et al., 2007). For each of these models, we requested 10,000 random sets of starting values each with 100 iterations and retained the best 100 solutions for final stage optimization (Meyer and Morin, 2016). The statistical adequacy of models relative to each other can be examined using a combination of relative fix indices [Akaike Information Criteria (AIC)]; Bayesian Information Criteria (BIC) and its sample size-adjusted version (ABIC); consistent AIC (CAIC), ratio test [Lo-Mendell-Rubin likelihood (LMR) and Bootstrap likelihood (BLRT) with 200 draws to estimate the *p*-value of the test]; and an indicator of the precision of class allocation (entropy). Lower values on the relative fit indexes, a statistically significant ratio test, and entropy values that are closest to one and larger in comparison to other profile structures provide evidence for a better fitting model (Nylund et al., 2007). Guided by simulation evidence (e.g., Peugh and Fan, 2013) and recent empirical work (e.g., Gabriel et al., 2018), we prioritized the BIC, ABIC, CAIC, and BLRT to identify the optimal model. As ratio tests are influenced heavily by sample size (Marsh et al., 2009; Morin et al., 2011), we also inspected graphical representations of relative fit indexes through “elbow plots” to identify the point at which the slope plateaus (Morin et al., 2020). We considered the statistical adequacy of solutions alongside their substantive meaning (e.g., congruence with theoretical perspectives) and profile membership (i.e., homogenous groups < 5% of total sample were considered spurious; Marsh et al., 2009). In the second step, we examined differences on hardiness between the latent subpopulations of cases (i.e., the optimal solution identified in step one) using multinomial regression within an LPA framework to assess outcomes of latent profile membership on hardiness; specifically, the BCH command modeled hardiness as an outcome of latent profile membership using weights to capture measurement error (Asparouhov and Muthén, 2018). A key strength of multinomial regression within an LPA framework is that auxiliary variables (hardiness in our case) are excluded from the classification model, yet their differences between sub-populations take into consideration most likely class membership and classification error (Morin et al., 2011; Wang and Hanges, 2011). We performed the primary analyses with M*plus* 8.2 (Muthén and Muthén, 2017).

### Results

Correlations among factor scores and the composite reliabilities are provided in Table 1.

**TABLE 1:** Standardized correlations among factor scores and their estimate of internal reliability (ω).

		**1**	**2**	**3**	**4**	**5**	**6**	**7**
1	Engage in self-reflection^a^	(0.82)						
2	Need for self-reflection^a^	0.96	(0.84)					
3	Insight^a^	0.31	0.14	(0.73)				
4	Habitual action^b^	–0.19	–0.14	0.06	(0.69)			
5	Understanding^b^	0.58	0.58	0.43	0.22	(0.57)		
6	Reflection^b^	0.65	0.58	0.39	0.10	0.86	(0.78)	
7	Critical reflection^b^	0.54	0.58	0.17	0.17	0.78	0.69	(0.71)

*^*a*^Subscale of the SRIS. ^*b*^Subscale of the QRT. Omega is presented on the diagonal in parentheses; all values > ± 0.11 were statistically significant at p < 0.01.*

An overview of the fit indices for the LPA models is provided in Table 2.

**TABLE 2:** Fit statistics of latent profile analyses.

							**LMR**	**BLRT**	
	**LL**	**FP**	**AIC**	**BIC**	**ABIC**	**CAIC**	***p*-value**	***p*-value**	**Entropy**
1-class	−1, 988.92	14	4,005.83	4,061.78	4,017.36	4,075.78	–	–	–
2-class	−1, 458.14	29	2,974.28	3,090.18	2,998.16	3,119.18	<0.001	<0.001	0.910
3-class	−1, 206.56	44	2,501.12	2,676.96	2,633.00	2,720.96	0.014	<0.001	0.884
4-class	−1, 067.49	59	2,252.99	2,488.78	2,301.57	2,547.78	0.368	<0.001	0.907
5-class	–966.78	74	2,081.55	2,377.29	2,142.48	2,451.29	0.003	<0.001	0.910
6-class	–893.61	89	1,965.23	2,320.91	2,038.51	2,409.91	0.041	<0.001	0.904
7-class	–830.31	104	1,868.62	2,284.25	1,954.25	2,388.25	0.052	<0.001	0.916
8-class	–772.94	119	1,783.87	2,259.45	1,881.85	2,378.45	0.017	<0.001	0.921
9-class	–725.40	134	1,718.79	2,254.31	1,829.12	2,388.31	0.735	<0.001	0.909

*LL, log-likelihood; FP, free parameters; AIC, Akaike information criterion; BIC, Bayesian information criterion; CAIC, consistent Akaike information criterion (calculated as the number of free parameters plus the BIC value); ABIC, sample-size-adjusted BIC; LMR, Lo et al. (2001) adjusted LRT test; BLRT, bootstrapped loglikelihood ratio test. All profiles were modeled in which the means and variances per profile indicator were allowed to vary across profiles. MLR, BLRT, and entropy statistics are unavailable when only 1 profile is calculated.*

**FIGURE 2:**
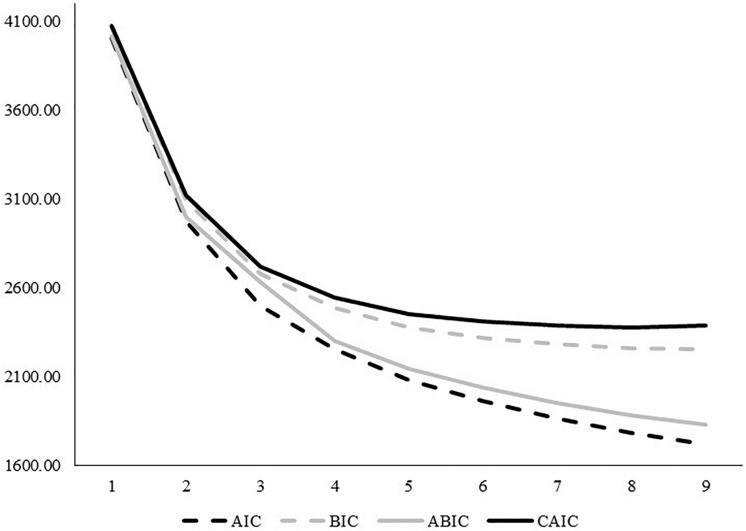
Elbow plot of the information criteria for the latent profile analysis.

The information criteria supported the ongoing addition of profiles to the solution without reaching a minimum value, with the exception of the CAIC. Visual inspection of the elbow plot showed that the improvement in fit flattens around four profiles for the CAIC and BIC and six profiles for the ABIC and AIC (see Figure 2). All of these profiles (four, five, and six sub-groups) were statistically sound, with each containing at least 8% of the total sample. The move from a four-profile to five-profile solution included qualitatively distinct sub-groups (i.e., each group is characterized by different configurations in the magnitude of the profile indicators), whereas the move from a five-profile to six-profile solution suggested that one sub-group divided into two with little meaningful distinction between them. Thus, we opted for the five-profile model solution.

**FIGURE 3:**
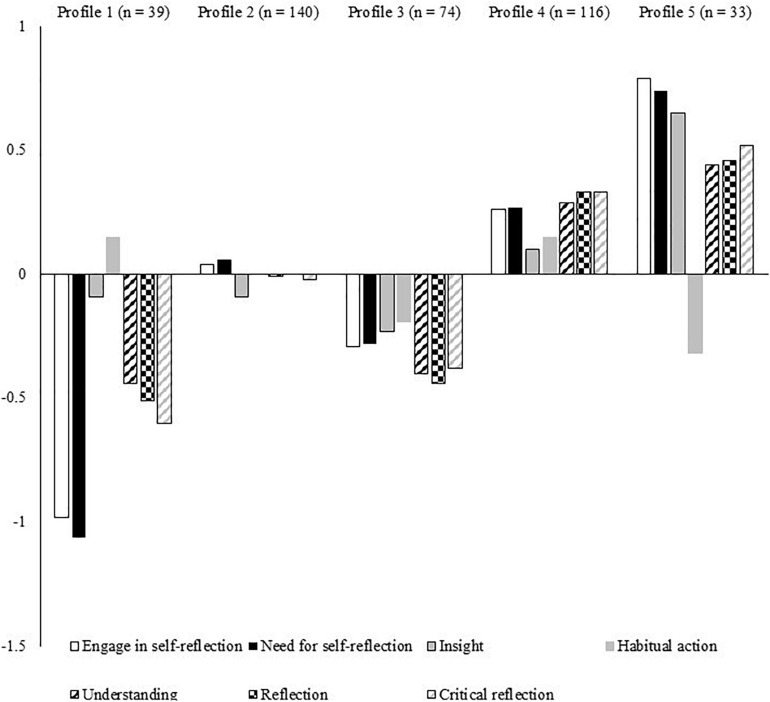
Final latent profile solution. Profile 1, highly disengaged, non-reflective, habitual actors; Profile 2, moderately engaged, surface level reflective thinkers; Profile 3, disengaged, non-reflective thinkers; Profile 4, engaged reflective thinkers; Profile 5, highly engaged, intentionally critical reflective thinkers.

In terms of standardized factor scores (see Figure 3), the largest profile (*n* = 140, 35%) encompassed individuals who reported average levels across all seven reflection indicators. We labeled these coaches *moderately engaged, surface-level reflective thinkers*. The second largest profile (*n* = 116, 29%) represented individuals who reported low-to-moderately high levels of all seven reflection indictors, with the exception of insight and habitual understanding, where they assessed themselves as relatively average. We labeled these coaches *engaged reflective thinkers*. The third largest profile (*n* = 74, 18%) included individuals who reported moderately low levels of all seven reflection indictors. We labeled these coaches *disengaged, non-reflective thinkers*. The second smallest profile (*n* = 39, 10%) was characterized by individuals who reported moderate-to-large low levels of all seven reflection indictors, with the exception of insight and habitual understanding, where they assessed themselves as relatively average. We labeled these coaches *highly disengaged, non-reflective, habitual actors*. The smallest profile (*n* = 33, 8%) captured individuals who reported high levels of all seven reflection indicators, with the exception of habitual understanding, where they reported moderately low levels. We labeled these coaches *highly engaged, intentionally critical reflective thinkers*.

To understand the nature of profile membership, we examined differences on hardiness between the five latent subgroups (see Table 3). A visual inspection of the variable means indicates that the *highly engaged, intentionally critical reflective thinkers* (profile 5) reported the highest level of all three hardiness facets, whereas the *highly disengaged, non-reflective, habitual actors* (profile 1) reported the lowest level of all three dimensions. Hardiness scores for the other three profile groups were largely similar in magnitude and situated between the extremes of profiles 1 and 5. The inferential tests supported the adaptive nature of profile 5, such that participants in this profile reported statistically higher levels of all three hardiness dimensions than all other subgroups. In contrast, the *highly disengaged, non-reflective, habitual actors* (profile 1) reported statistically lower levels of control than all other profiles, though the comparisons were mixed for commitment and challenge. The *moderately engaged reflective thinkers* (profile 4) also reported generally higher levels of all three hardiness dimensions relative to the other subgroups. Collectively, these findings indicate that the most meaningful differences between profiles with regard to hardiness scores are observed when individuals reported low or moderate-to-high levels of most reflection dimensions.

**TABLE 3:** Latent profile differences in hardiness including means, standard deviations, and Cohen’s *d* (*n* = 402).

	***M***	***SD***	**P1**	**P2**	**P3**	**P4**	**P5**
			**Commitment**				

Profile 1	10.37	2.81	na				
Profile 2	10.90	2.96	–0.18	na			
Profile 3	10.68	2.49	–0.12	0.08	na		
Profile 4	11.80	2.69	−0.52*	−0.32*	−0.43*	na	
Profile 5	13.23	1.44	−1.35*	−1.06*	−1.30*	−0.69*	na

			**Control**				

Profile 1	10.28	2.56	na				
Profile 2	12.12	2.48	−0.73*	na			
Profile 3	11.89	2.24	−0.67*	0.10	na		
Profile 4	12.09	2.69	−0.69*	0.01	–0.08	na	
Profile 5	13.57	1.84	−1.50*	−0.67*	−0.82*	−0.65*	na

			**Challenge**				

Profile 1	7.88	3.06	na				
Profile 2	9.32	2.96	−0.48*	na			
Profile 3	8.19	3.01	–0.10	0.38*	na		
Profile 4	9.10	2.91	−0.41*	0.08	−0.31*	na	
Profile 5	11.48	2.01	−1.42*	−0.87*	−1.31*	−0.97*	na

Profile 1 (P1), highly disengaged, non-reflective, habitual actors; Profile 2 (P2), moderately engaged, surface level reflective thinkers; Profile 3 (P3), disengaged, non-reflective thinkers; Profile 4 (P4), engaged reflective thinkers; Profile 5 (P5), highly engaged, intentionally critical reflective thinkers; numerical values represent Cohen’s d (e.g., standardized mean difference between profiles 1 and 5 on commitment is −1.35); na, not applicable; *p < 0.05.

## General Discussion

Through the two studies presented in this manuscript, we aimed to examine how sports coaches might develop and optimize hardiness, a form of dispositional resilience that is thought to be influential to the way individuals manage demanding situations (Hanton et al., 2013; Mazerolle et al., 2018). In the first study, we interviewed elite coaches who were identified as high hardy about the critical incidents they had experienced that resulted in such dispositions. While a number of developmental factors were identified (e.g., external support, coping mechanisms, exposure to different experiences), a key process that was proposed to be common to the development of hardiness across the different themes was RP. Specifically, participants identified how RP facilitated a process of experiential learning that resulted in greater self-awareness, insight, and the development of adaptive behaviors (including the use of different coping mechanisms), which were thought to enhance levels of hardiness. In the follow-up study, we explored this relationship (e.g., RP and hardiness) further by investigating whether an individual’s RP profile (e.g., type of engagement, level of reflection) was associated with differing levels of hardiness. Using multinomial regression within an LPA framework, we found that the profile of participants who demonstrated purposeful engagement and the ability to reflect more critically had significantly higher levels of each of the sub-components of hardiness than those in all other profile sub-groups. Thus, we provide novel insights into the development of a hardy disposition and its attitudinal sub-components and into the potentially vital role that individuals’ RP tendencies plays in optimizing coach hardiness.

Khoshaba and Maddi (1999) observed that hardiness and associated coping strategies develop over time and through confronting adverse experiences in a functional manner (e.g., engaging in goal-directed, approach-based behaviors). The findings of our research (Study 1) support this perspective, with participants recognizing the developmental benefits of experiencing and learning from a range of different difficult situations within different contextual frames (e.g., demands, adversity, context-specific). For example, of particular relevance to the participants in Study 1, were the opportunities to develop their hardy dispositions through *critical sporting incidents*, as these experiences were thought to help them better understand the demands and what was required to manage them on a daily basis in their work lives. Similarly, Mazerolle et al. (2018) referred to the importance of individuals developing the beliefs and attitudes required to successfully acclimatize and thrive within the context of a given work role. Facilitating early exposure to the specific environments and demands that a coach will experience in their roles, coupled with appropriate support, may therefore be beneficial in supporting the growth of hardy dispositions (cf. Bartone et al., 2016; Fletcher and Sarkar, 2016). Based on our findings, however, we suggest that mere exposure to different demands is insufficient to facilitate adaptive responses and hardiness development. Experiences have to, instead, be transformed into learning through RP (Peel et al., 2013; Cropley et al., 2018). Indeed, RP, conducted in a meaningful and critical way, is thought to reinforce learning, augment critical thinking and self-discovery, aid the development of new meaning, and support personal and professional growth (Marie et al., 2017; Cropley et al., 2018; Crane et al., 2019). RP may, therefore, facilitate developments in hardiness in a number of ways. First, it is widely reported that a product of RP is improved self-awareness, which, in turn, is considered as an influential psychological process that facilitates positive outcomes and adaptive responses to stressful situations (Cowden and Meyer-Weitz, 2016). This contention is substantiated in our research (Study 2) with the sub-group of participants who demonstrated the highest levels of hardiness also reporting higher levels of insight (i.e., self-awareness). Second, we argue that the learning that emerges from RP can elicit adaptive actions, such as gaining access to resources that are necessary for coping (Crane et al., 2019). In support of this proposal, the importance of certain experientially developed coping mechanisms to enhance hardiness, was displayed in our first study. Third, the effortful cognitive processing, sense making, and action planning required as part of the RP process can help individuals to reappraise the situations/problems that they have experienced. In doing so, the RP process is thought to direct individuals to commit to positive future action and improve their sense of control over future situations as they seek to implement the deep learning gleaned from their RP (Faull and Cropley, 2009; Hanton et al., 2009). Finally, the purposeful and critical use of RP underpins the hardiness sub-components as it encourages individuals to identify new experiences as opportunities for learning and personal growth, and then to plan effectively to manage future experiences (control), which provides a sense of commitment to achieve (Knowles et al., 2012; Stein and Bartone, 2020). It would appear, therefore, that RP enables individuals to elicit the learning from experience that supports the management of demanding and unpredictable circumstances and results in more adaptive responses to challenges.

The findings of our second study support the potential importance of RP as a meta-cognitive strategy that can optimize hardiness in coaches. However, these findings also detail the key role that the level of an individual’s RP plays in their experience of hardiness and its sub-components. For example, higher levels of hardiness were found in those participants who reported a highly engaged and critically reflective profile than in those who reported disengaged, non-reflective profiles. There has been considerable debate in the RP literature concerning the nature and importance of critical levels of reflection (e.g., Knowles and Gilbourne, 2010; Cushion, 2018). The authors are in agreement, however, that critical reflection is both emancipatory (i.e., it sets individuals free from constraining influences) and transformational (i.e., it results in enlightenment and empowerment to improve thoughts and behaviors; Cropley et al., 2020a). Conversely, lower levels of reflection are more concerned with issues of the efficiency of, and accountability to, practice (e.g., exploring whether certain actions achieved the desired outcome). Thus, critical reflection is considered to be a deeper, more thoughtful and more profound level of reflection that requires a change of perspective and alterations to deeply held beliefs (Legare and Armstrong, 2017). Such a view helps to substantiate the importance of critical reflection, as reported in Study 2, for the development of hardiness given the need for adaptive, transformative processes to improve perceptions of control, commitment, and challenge. For example, in Study 2, the sub-group of *engaged reflective thinkers* (profile 4) demonstrated significantly lower levels of hardiness than the sub-group of *highly engaged, intentionally critical reflective thinkers* (profile 5), but higher overall hardiness than the other profiles (who saw the need for, and engaged in, reflective thinking less). As a result of this finding, we suggest that engagement in RP is necessary, yet an individual’s ability to engage in higher-order reflective thinking is potentially most important in relation to the development of hardiness.

The LPA adopted in Study 2 indicated that the two largest sub-groups of participants were *moderately engaged, surface level reflective thinkers*, and *engaged reflective thinkers* respectively, with the smallest sub-group of participants being the *highly engaged, intentionally critical reflective thinkers*. These sub-groups also indicated higher levels of overall hardiness than the two *non-reflective* sub-groups. These findings suggest two things: (a) a large proportion of our sample reported a need to reflect and at least some engagement in RP; and (b) only a small proportion of the sample reflect at the critical levels required to achieve greater developmental adaptations to hardiness and its sub-components. In support of this, researchers have argued that critical RP is difficult to attain, requiring a specific mindset and commitment to the process (Crane et al., 2019). Perhaps as a result, much of what is currently considered (and practiced) as meaningful (critical) reflection in coaching has been labeled as little more than descriptive evaluation of practice (Cushion, 2018). Further, previous research has reported that many coaches (particularly neophyte) experience a lack of confidence in their understanding of RP, which contributes to limited engagement with the process (Burt and Morgan, 2014). From an applied perspective, therefore, in considering the development of hardiness and its attitudinal sub-components, there is a need for practitioners (e.g., sport psychologists, coach educators) to promote engagement in RP and also develop coaches’ confidence and ability to reflect at critical levels of insight, which can be done through systematic training and support programs (Faull and Cropley, 2009; Bulman et al., 2016). Indeed, stress management training programs that focus on longer-term hardiness development may be supplemented by encouraging greater self-awareness, personal growth, and adaptive responses through more meaningful and critical RP (cf. Crane et al., 2019).

### Limitations and Future Directions

Focusing on the limitations of our research, while the participants in Study 1 were carefully selected based on criteria designed to ensure an information rich sample (i.e., high hardy), we cannot presume that sub- or non-elite coaches would not also demonstrate high levels of hardiness and, therefore, provide very different insights into the development of hardy dispositions. In Study 2, however, we did sample coaches working at all levels of their respective sports, identifying participants across those levels who profiled into each of the sub-groups. Given that the majority of the sport coaching workforce operate at sub-elite levels, researchers may wish to explore potential comparisons between elite and non-elite coach hardiness, as well as investigate whether RP profiles differ across coaches working at various levels of performance. Indeed, researchers have detailed how coaches operating at all levels experience considerable, yet varying demands (e.g., Potts et al., 2018). Such future research would, therefore, help to further understanding about how well coaches are prepared to function effectively at their respective level, and offer insights into how to better educate and support coaches to develop the attitudes associated with hardiness. In addition, a distinct gender bias was observed in the sample for both studies. While we actively encouraged participation in attempts to gain a more balanced gender split, we were not able to properly address this within the data collection period. Researchers are encouraged, therefore, to achieve a gender-balanced sample in future examinations of hardiness development and the role of RP in facilitating hardy attitudes. In doing so, a wider understanding of the potential nuances relating to RP, hardiness, and gender within sport coaching can be obtained.

By adopting a critical incident approach in Study 1, we focused on three key experiences thought to have influenced participants’ hardiness. While the semi-structured nature of the interviews afforded us the opportunity to explore beyond these experiences, we appreciate that other frameworks have been developed to guide temporal enquiry (e.g., event temporal sequencing; Salim et al., 2016) and that more concurrent forms of investigation may be beneficial to track the development of hardiness over time. Finally, the findings from both studies are based on coaches’ self-reports. While every effort was made to request and facilitate honest responses, there is always the likelihood of socially desirable responding or self-report bias. However, by utilizing a person-centered approach to analysis (e.g., LPA) in Study 2, we produced unique insights into clusters of participants who shared similar RP profiles (e.g., engagement in, and ability to, reflect). We are the first group of researchers to have explored profiles of RP indices. Consequently, researchers should consider further investigating of the role that RP plays in supporting the development of hardy dispositions, particularly in relation to the level of RP individuals tend to engage with.

## Conclusion

Hardiness is widely associated with desirable health- and performance-related outcomes because it is considered as a personal characteristic that helps individuals to transform debilitating situations into opportunities for personal growth (Salim et al., 2016). The development of hardy dispositions in those working in demanding environments is, therefore, efficacious. This propensity is particularly true for sports coaches who regularly experience a range of personal, performance, and organizational demands that have the potential to increase the likelihood of burnout (Bentzen et al., 2017) and reduce performance effectiveness (Thelwell et al., 2017). In light of this, the findings of our research provide unique insights into the role of a number of mechanisms that support increases in hardiness and its sub-components. Of particular significance was the participating coaches’ RP profiles (e.g., type of engagement, level of reflection), which were found to be directly linked to higher levels of hardiness. As a result, our findings offer support for the contention that hardiness can be developed, particularly through focusing on each of its sub-components, but not necessarily through exposure to demands alone. Coaches need to engage in critical levels of RP in order to transform demanding experiences into the learning required to elicit adaptive responses and thus gradually develop their hardy dispositions over time. However, additional support for our novel findings and contentions, through longitudinal examination of the relationship between RP and hardiness in coaches and wider sport support staff, is warranted.

## Data Availability Statement

The datasets generated for this study are available on request to the corresponding author.

## Ethics Statement

Both studies were reviewed and approved by the Faculty of Life Sciences Research Ethics Committee at the University of South Wales (approval code: 180901AMLR). Accordingly, our research was conducted in compliance with the principles of the Declaration of Helsinki and written informed consent to participate was provided by all participants in both studies.

## Author Contributions

BC, LB, SH, and RN were involved in the conceptualization of the research question and study design. LB was responsible for data collection in study one. LB and AM conducted the study one data analysis and construction of the findings (entire research team acted as critical friends). DG conducted the study two data analysis and preparation of findings. BC, LB, RN, and TW prepared the manuscript in its entirety with SH and DG responsible for manuscript editing. All authors were involved in data collection for study two.

## Conflict of Interest

The authors declare that the research was conducted in the absence of any commercial or financial relationships that could be construed as a potential conflict of interest.

## Footnotes

1 It should be noted that while hardiness and resilience are related (e.g., hardiness represents a psychological disposition that can be developed to increase resilience; Stein and Bartone, 2020), they are considered as distinct constructs. To distinguish the constructs further, *resilience* is best viewed as an outcome that emerges over time via dynamic person–situation interactions, and either maintains functioning, or returns it to normal levels after a level of deterioration, following exposure to adversity (Gucciardi et al., 2018).
